# Effect of preventive zinc supplementation on linear growth in children under 5 years of age in developing countries: a meta-analysis of studies for input to the lives saved tool

**DOI:** 10.1186/1471-2458-11-S3-S22

**Published:** 2011-04-13

**Authors:** Aamer Imdad, Zulfiqar A Bhutta

**Affiliations:** 1 Division of Women and Child Health, Aga Khan University, Karachi, Pakistan

## Abstract

**Introduction:**

Zinc plays an important role in cellular growth, cellular differentiation and metabolism. The results of previous meta-analyses evaluating effect of zinc supplementation on linear growth are inconsistent. We have updated and evaluated the available evidence according to Grading of Recommendations, Assessment, Development and Evaluation (GRADE) criteria and tried to explain the difference in results of the previous reviews.

**Methods:**

A literature search was done on PubMed, Cochrane Library, IZiNCG database and WHO regional data bases using different terms for zinc and linear growth (height). Data were abstracted in a standardized form. Data were analyzed in two ways i.e. weighted mean difference (effect size) and pooled mean difference for absolute increment in length in centimeters. Random effect models were used for these pooled estimates. We have given our recommendations for effectiveness of zinc supplementation in the form of absolute increment in length (cm) in zinc supplemented group compared to control for input to Live Saves Tool (LiST).

**Results:**

There were thirty six studies assessing the effect of zinc supplementation on linear growth in children < 5 years from developing countries. In eleven of these studies, zinc was given in combination with other micronutrients (iron, vitamin A, etc). The final effect size after pooling all the data sets (zinc ± iron etc) showed a significant positive effect of zinc supplementation on linear growth [Effect size: 0.13 (95% CI 0.04, 0.21), random model] in the developing countries. A subgroup analysis by excluding those data sets where zinc was supplemented in combination with iron showed a more pronounced effect of zinc supplementation on linear growth [Weighed mean difference 0.19 (95 % CI 0.08, 0.30), random model]. A subgroup analysis from studies that reported actual increase in length (cm) showed that a dose of 10 mg zinc/day for duration of 24 weeks led to a net a gain of 0.37 (±0.25) cm in zinc supplemented group compared to placebo. This estimate is recommended for inclusion in Lives Saved Tool (LiST) model.

**Conclusions:**

Zinc supplementation has a significant positive effect on linear growth, especially when administered alone, and should be included in national strategies to reduce stunting in children < 5 years of age in developing countries.

## Introduction

The association of zinc deficiency with growth retardation and hypogonadism was first described in 1963 from Iran [1] and is now well established from animal and human studies demonstrating that zinc plays a critical role in cellular growth, cellular differentiation and metabolism [2].

Several studies have been conducted to explore the effect of zinc supplementation on children’s growth. Although there have been several reviews published on this topic, to date few meta-analyses have been published in this regard [3-5]. The two meta-analyses by Brown et al. [3,4] that included studies of zinc supplementation in pre-pubertal children concluded that zinc supplementation produces highly significant positive effect on height gain. In contrast, a recent meta-analysis by Ramakrishnan et al. [5] concluded that zinc supplementation was not associated with any significant positive effect on linear growth in children < 5 years of age.

We have reviewed the available literature and evaluated the quality of included studies according to Child Health Epidemiology Reference Group (CHERG) adaptation of Grading of Recommendations, Assessment, Development and Evaluation (GRADE) criteria [6,7]. We have updated the previous meta-analyses and have tried to explore and explain the difference in results of the above mentioned meta-analyses. We have also generated an estimate for actual increase in length (cm) for input into Lives Saved Tool (LiST) model for a standard dose and duration of zinc supplementation in children < 5 years in developing countries.

## Methods

### Searching

To evaluate the effect of zinc supplementation on linear growth, we conducted a literature search on PubMed, Cochrane Library, IZiNCG database and WHO regional databases library as well as a manual check of available reviews and previous meta-analyses on the subject. There was no language restriction. The following search strategy was used for literature search: (“growth” OR “height” OR “weight” OR “stunting” OR growt* OR heigh* OR weigh* OR stuntin*) AND (“Zinc” OR “Zinc sulphate”) and limited to “clinical trial” and “humans”. The last date of search was March 3rd 2010.

### Inclusion/exclusion criteria

The following inclusion criteria were used to identify the studies for data abstraction: a) the study was a randomized, placebo-controlled intervention trial conducted in a developing country b) the subjects were children less than 5 year of age c) the subjects were not premature infants; d) the subjects were free of chronic diseases, such as sickle cell disease, cystic fibrosis, or severe protein-energy malnutrition e) zinc was the only component of the supplement that differed between treatment groups and f) the supplemental zinc was provided for at least ≥8 weeks. There was no restriction on dose of zinc supplemented or formula used (e.g. Zinc sulphate or Zinc Gluconate etc). The developing countries were defined as countries with Gross National Income per capita (GNI) below US$11,905, according to World Bank [8]. We excluded studies where zinc was provided in fortified food as this was considered a different intervention that has been reviewed elsewhere [9].

### Data abstraction and validity assessment

For quality assessment, data were abstracted into standardized forms using variables as study identifiers and context, study design and limitations, intervention specifics, and outcome effects [7]. Two authors entered the data and discrepancies were removed, if found. Individual studies were graded according to strengths and limitations of the study. Studies received an initial score of high if a randomized or cluster randomized trial and then the grade was decreased for each study design limitation, if applicable. A study was downgraded if there were limitations in the conduct of studies e.g. inadequate methods of sequence generation or allocation concealment and/or high loss to follow up (>20%). Risk of bias in the included studies was assessed according to latest Cochrane handbook and findings are presented in Additional File 1. Finally each study was assigned a final quality grade of “high” “moderate” “low” or “very low” on the basis of strengths and limitations of study [6,10]. Studies receiving a grade of ‘very low’ were excluded from the analysis. The grading of overall (pooled) evidence was based on three components: (1) the volume and consistency of the evidence; (2) the size of the pooled effect and (3) the strength of the statistical evidence reflected in the p-value [10]. A similar grading of ‘high’ ‘moderate’ ‘low’ and ‘very low’ was used for grading the *overall* evidence indicating the strength of an effect of the intervention on specific health outcome [10].

### Quantitative data synthesis

The primary outcome was change in height [expressed in cms or height-for-age Z score (HAZ)]. For studies where mean change in height was not reported, it was calculated as the difference of mean post- and pre-intervention measurements. If studies did not report the standard deviation (SD) for change in height, it was calculated assuming that the correlation between the pre- and post-test variances was equal to the average correlation found in available studies. If studies reported the standard error (SE), we calculated SD by multiplying SE with square root of sample size. For studies that had different sample sizes at the beginning and the end of the intervention, the lower value of the two was used in analysis. The assessment of statistical heterogeneity in the pooled analysis was done by visual inspection i.e. the overlap of the confidence intervals among the studies, Chi square (P-value) of heterogeneity in the meta-analyses and I^2^ statistics. A low P value (less than 0.10) or a large chi-squared statistic relative to its degree of freedom and I^2^ values greater than 50% were taken as substantial and high heterogeneity. In situations of substantial or high heterogeneity being present, causes were explored by sensitivity analysis and random effects model were used.

In studies with factorial design i.e. (two or more intervention comparisons carried out simultaneously in a single study), only data in which zinc was the only difference between the two groups were included. For example, for iron-zinc factorial trials, results were included for comparisons of zinc only vs. placebo and zinc plus iron vs. iron only. In case of cluster randomized trials, cluster adjusted values were used.

Data were analyzed in two ways. In first analysis, we pooled the studies to get a weighted mean difference also called effect size. This value, also known as Cohen’s effect size, is useful in meta-analyses because it eliminates the problems of units of measurement (e.g. change in height in cm or HAZ scores) and duration, which may vary across studies [11,12]. These effect sizes were calculated for individual studies by dividing the difference between the mean change in treatment and control groups by the pooled standard deviation. Random effect models were used for the primary analysis [13]. We did a subgroup analysis for data sets where zinc was supplemented alone by excluding those data sets where it was given in combination with iron. This was based on results of some experimental studies that had shown that iron may decrease the absorption of zinc when supplemented together [14,15]. We hypothesized that the preventive effect of zinc for stunting would be more prominent if supplemented alone rather than in combination with iron.

In the second analysis, we pooled the studies reporting change in height in cms to get a net change in length in intervention group compared to control. We also did a *post hoc* subgroup analysis for different daily dosages of zinc supplementation to get a point estimate for inclusion in the Live Saved Tool (LiST). More details about this analysis are provided in the results section (Recommendations for LiST model). All the meta-analyses were conducted using software Review Manager version 5 [16].

## Results

### Trial flow

**Figure 1:**
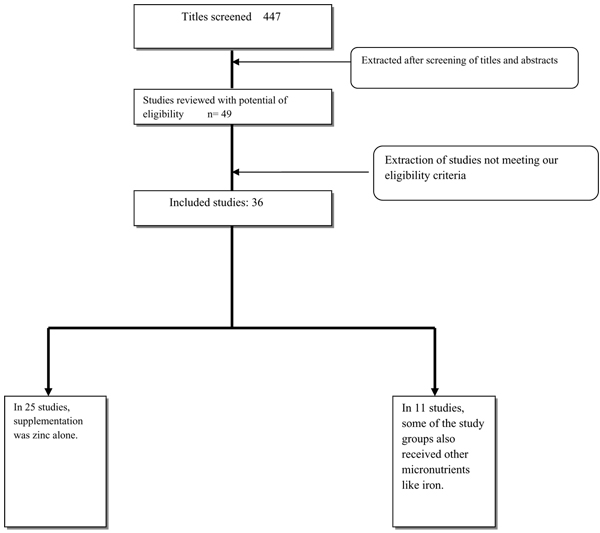
Synthesis of study identification in review of the effects of zinc supplementation on growth.

From literature search, 447 titles were identified (Fig: 1). We scanned the titles and abstracts of the trials identified to exclude those that were obviously irrelevant, retrieved the full text of the remaining trials, and identified relevant articles. Initially, 49 studies were selected for detailed review. Five of these studies were not included in the final analysis because the participants were > 5 years of age [17-22]. Four studies were excluded from analysis because they were from developed countries [23-26], as per objectives of LiST model. Two studies were excluded because the duration of supplementation was < 8 weeks [27,28]. One study was excluded because it included only premature babies [29]. Three studies were excluded because children had severe protein energy malnutrition [30-32]. Finally, 36 studies were selected for data abstraction [33-68].

### Study characteristics

Table 1 presents the characteristics of included studies. All the included studies were randomized controlled trials. In 25 studies zinc was supplemented alone while in 11 studies some of the study groups also received other micronutrients (iron, folic acid, vitamin A) [47,51,52,54,58,59,61-63,65,68]. Mean initial height for age Z score (HAZ) ranged from -2.91 [37] to -0.08 [33], and the mean initial age ranged from less than one month [50] to 55 months[40]. The average daily dose (calculated by dividing the total weekly doses by 7) of zinc supplementation ranged from 1 mg/day [41] to 20 mg/day [53] with a median of 10 mg/day. Most studies (*n* = 28) provided the zinc supplements in the form of zinc sulphate, although 5 used zinc acetate [36,44,48,53,56] and 2 used zinc gluconate [34,55]. The mean duration of intervention ranged from 8 weeks [41] to 64 weeks [35] with a median of 24 weeks. All the included studies were published between 1992 and 2009 (median: 1998). Fourteen of these studies were conducted in Latin America or the Caribbean, 16 in Asia, and 6 in Africa. Additional File 1 presents the risk of bias table for included studies.

**Table 1:** Characteristics of included studies

Study ID (ref)	Country	Sample size (*n*)	Mean initial age (*months*)	Dose (*mg/day*)	Duration (*weeks*)	Mean initial height (*cm*)	Mean initial HAZ score	Grade (High, moderate and low)
Hong 1992[33]	China	102	0	5	24	50.0	-0.08	Moderate
Bates 1993[34]	Gambia	110	18	70 (/2wk)	64	76.8	-1.57	High
Dirren 1994[35]	Ecuador	96	32	10	64	81.6	-2.89	Moderate
Castillo-Duran 1995[36]	Chile	68	0	3	24	47.2	-1.36	Moderate
Ninh 1996[37]	Vietnam	146	17.5	10	20	71.3	-2.91	Moderate
Sempertgui 1996[38]	Ecuador	50	42.3	10	9	_	-2.00	Moderate
Ruz 1997[39]	Chile	102	39.8	10	56	95.6	-0.52	High
Rosado 1997	Mexico	109	28	20	52	83.4	-1.55	moderate
Gardner 1998[42]	Jamaica	61	14	10	12	68.7	-2.85	High
Lira 1998[41]	Brazil	137	0	1 and 5	8	_	_	Moderate
Kikafunda 1998[40]	Uganda	155	55.8	10	24	103.3	-0.70	Moderate
Rivera 1998[43]	Guatemala	89	7.5	10	28	63.7	-2.16	High
Smith 1999[44]	Belize	51	44	70	24	_	_	High
Umeta 2000 [45]	Ethopia	100	9.6	10	24	69.9	-0.64	High
Castillo-Duran 2001[46]	Chile	150	0	5	52	50.3	-0.06	High
Dijkhuizen 2001[47]	Indonesia	238	4.2	10	24	61.2	-0.79	High
Osendarp 2002[48]	Bangladesh	301	0.9	5	20	51.2	0.00	Low
Muller 2003 [49]	Burkina Faso	709	18.5	24	75.5	8.7	-1.55	High
Sur 2003[50]	India	100	0	5	52	46.4	-1.70	High
Black 2004[52]	Bangladesh	94	6.5	20	24	_	-1.20	Moderate
Black 2004[53]	India	200	1	5	32	47.5	-1.20	Moderate
Lind 2004[54]	Indonesia	340	6	10	24	_	-0.35	High
Alarcon 2004[51]	Peru	213	17	3	18	76.8	-1.04	High
Penny 2004[55]	Peru	246	19	10	24	76.4	-0.16	High
Gardner 2005[57]	Jamaica	114	19	10	24	77.1	-1.65	Moderate
Brooks2005[56]	Bangladesh	1665	5.3	70(/wk)	52	62.7	-1.10	High
Wasantwist 2006[61]	Thailand	304	4.5	10	24	62.3	-0.70	High
Silva 2006 [60]	Brazil	58	23.5	10	16	_	-1.95	High
Berger 2006[58]	Vietnam	391	5.9	10	24	63.8	-1.07	High
Olney 2006[59]	Tanzania	433	8.8	10	52	_	-1.45	Moderate
Brown 2007[67]	Peru	302	7.5	3	24	65.4	-1.19	High
Fahmida 2007[62]	Indonesia	399	5	10	24	_	-0.99	High
Wuehler 2008[64]	Ecuador	253	21	3,7and10	24	77.4	-2.30	High
Dijikhuizen 2008[63]	Multicentre trial (Thailand, Veitnam and Indonesia)	2468	5.05	10	24	_	-0.82	High
Mozaffari-Khosarvi 2009[66]	Iran	85	38.8	5	24	_	-1.65	High
Walker 2009 [65]	Bangladesh	645	6.35	20	24	64.2	_	High

### Effect on linear growth

Information on change in height (in cm or HAZ scores) was available from 35 studies, which contained 47 group wise comparisons. In study by Fahmida et al. 2007 [62], only one data set was included in analysis (zinc alone vs. placebo) because comparison groups for other data sets (zinc + iron, zinc + iron + vitamin A) were not appropriate (no iron only or iron + vitamin A group). In study by Brown et al. 2007 [67], there were three intervention groups i.e. placebo, zinc alone (solution form) and zinc (in fortified food). We included comparison of zinc (solution form) vs. placebo and not that of zinc in fortified food vs. placebo. Iron was supplemented to both groups in study by Alarcon et al. 2004 [51]. This study had been included in analysis of zinc + iron vs. iron alone and not that of zinc alone vs. control comparison.

**Figure 2:**
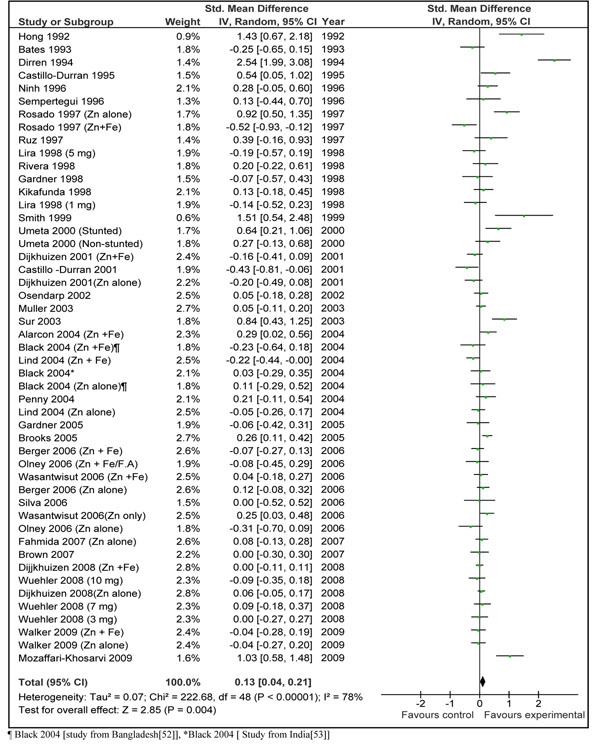
**Effect sizes for height gain in zinc intervention trials among children less than 5 years of age in developing countries. Final estimate from 48 data sets of 36 studies. (Includes data sets with zinc + iron)**[[Bibr B53]]

**Figure 3:**
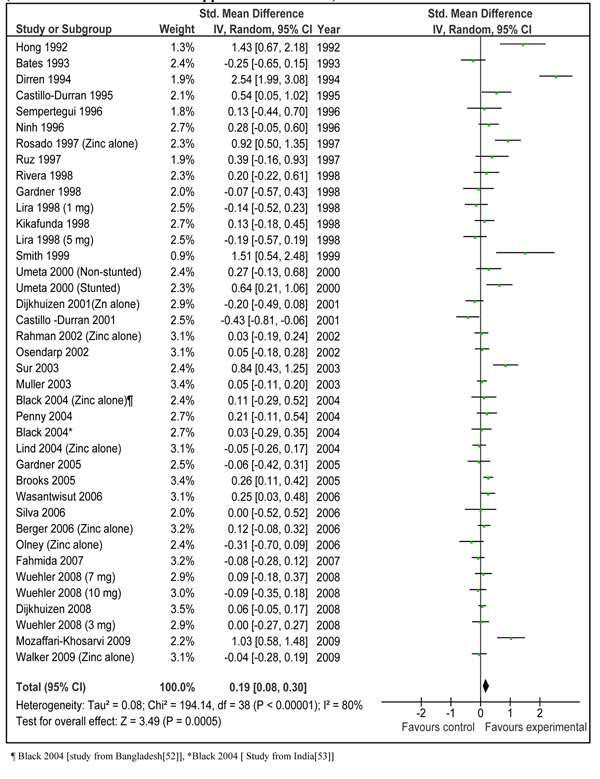
Effect sizes for height gain in zinc intervention trials among children less than 5 years of age in developing countries. Final estimate from 39 data sets of 34 studies. (Includes data sets with zinc supplementation alone)

Our meta-analysis of available studies suggested that zinc supplementation is associated with a net benefit on linear growth. The estimated effect size (weighed mean difference) for zinc supplementation on linear growth including results from all data sets (zinc ± iron) was 0.13 [95% CI 0.04, 0.21] (Figure 2). When data sets with zinc + iron were excluded from analysis, the final effect size was 0.19 [95 % CI 0.08, 0.30] (Figure 3). We also separately pooled results of data sets where zinc was given in combination with Iron (data not shown). The pooled estimate for zinc plus iron versus control was -0.10 [95 % CI -0.21, 0.01]. These results were significantly different from overall estimate (p=0.0001). This shows that zinc supplementation alone has more prominent effects on length gain than when supplemented in combination with iron. Due to this reason, we would base our recommendations for LiST model on the basis of results of zinc supplementation alone in the next section.

### Recommendations for LiST model

Results of effect size (weighed mean difference) are interpreted as the percent of non-overlap of the intervention group’s scores with those of the control group. An effect size (ES) of 0.0 indicates that the distribution of scores for the intervention group overlaps completely with the distribution of scores for the control group, and there is 0% non-overlap. An ES of 0.3 indicates a non-overlap of 21.3% in the two distributions. Effect size can be categorized as small (~0.2), medium (~0.5) or large (~0.8) [11]. This shows that results of pooled effect size can only be interpreted as percent of non-overlap of results of two groups and an absolute quantitative estimate cannot be generated in the form of units of measurement [69].

**Figure 4:**
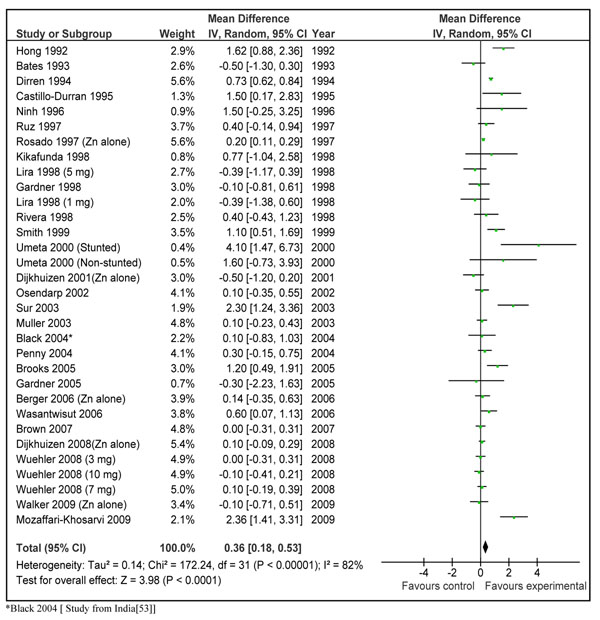
**Main gain in height (in cm) in zinc supplemented (alone) group compared to control. Data from 28 studies in the form 32 data sets irrespective of dose and duration of supplementation**.

**Figure 5:**
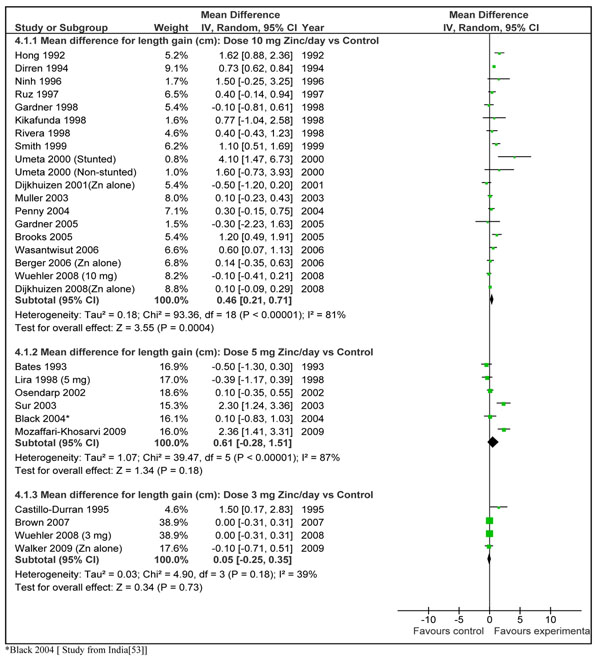
Forest plot for mean gain in height (cm) after zinc supplementation alone in children less than 5 years of age: Subgroup analysis according to different dosages

**Figure 6:**
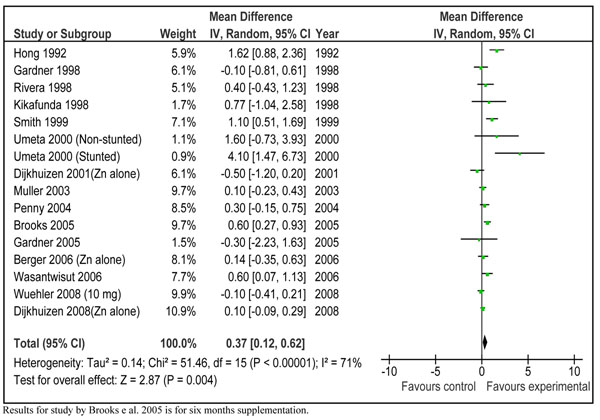
Mean gain in height (cm) after 10 mg zinc supplementation alone for 24 weeks in children < 5 years of age in developing countries

In order to translate the observed weighed mean effect size into practical recommendations, we reanalyzed the subset of 28 studies that presented results in terms of absolute height increments in centimeters. The pooled results from 32 data sets of these studies showed a net gain of 0.36 (±0.18) cm in the zinc supplemented group compared to control in children < 5 years of age in developing countries (Figure 4). The mean duration of supplementation in these studies was 7.03 months and the dose ranged from 1 mg/day to 20 mg/day. The weighed mean difference ‘effect size’ for these studies was 0.23 (95 % CI 0.11, 0.36). In order to give a recommendation with a specific *dose/day* and for a specific *duration*, we did a *post hoc* subgroup analysis according to different dosages of daily zinc supplementation (Figure 5). This analysis showed that preventive zinc supplementation in a *dose of 10 mg/day* has the most significant effect on linear length in children < 5 years of age [mean difference: 0.46cm (95 % CI 0.21, 0.71), random model]. The results for dose of 5 mg/day [mean difference 0.61 (95 % CI -0.28, 1.51), random model] and that of 3mg/day [mean difference 0.05 (95 % CI -0.25, 0.35) random model] were not statistically significant. There was a significant statistical difference among these subgroups (p=0.005). In the subset of studies where zinc was supplemented in a dose of 10 mg/day, duration of supplementation was 6 months in all the studies except in three studies [35,37,39]. In two of these studies the supplementation continued for a year [35,39] while in one study it was for 5 months [37]. In the study by Brooks et al. [56] the supplementation also continued until a year, however disaggregated data was available for 6 months duration. We pooled results of those studies where zinc was supplemented in a dose of 10 mg/day for duration of 24 weeks by excluding the three above mentioned studies [35,37,39]. Combined results from these studies showed a net gain in length of 0.37 cm (±0.25) in zinc supplemented group compared to placebo (Figure 6). Table 2 summarizes the quality assessment and pooled estimate for this outcome. The qualitative assessment of the available evidence for the effect of zinc supplementation (10 mg/day for 24 weeks) on linear growth was that of ‘moderate’ level. This qualitative grading for collective evidence is based on the parameters such as volume and consistency of the evidence, the size of the effect and the strength of the statistical evidence for an association between the intervention and outcome [10].

On the basis of qualitative grading of collective evidence and specificity of intervention in terms of dose and duration, we have recommended this estimate for input to LiST model. This can be described as follows; “preventive zinc supplementation in a dose of 10 mg/day for 24 weeks in children < 5 years of age leads to a net gain of 0.37cm in zinc supplemented group compared to control in developing countries”.

**Table 2:** Qualitative assessment of Trials for effect of zinc supplementation on linear growth

Quality Assessment						Summary of findings	
				Generalizability		Pooled Effect	Qualitative Assessment

No. of studies	Design	Limitations	Consistency	Generalizability to Population of Interest	Generalizability to intervention of Interest	Mean Difference ( 95 % CI)	High, moderate, low, very low)

Effect of Zinc supplementation on linear growth: Zinc supplementation for a dose of 10 mg/day for a duration of 24 weeks							

**15**[33,40,42-45,47,49,55-58,61,63,64]	RCT	In one study [49], dose was not exact 10 mg/day but was 12.5 mg/day. Supplementation in one study continued till 28 weeks [43].	Heterogeneity 71 %. Random effect models used.	All the studies from developing countries	10 mg zinc/day for 24 weeks	0.37(0.12-0.62) cm	Moderate

## Discussion

Effect of zinc supplementation on linear growth has been evaluated previously [3-5]. The first and most widely known meta-analysis evaluating the effect of zinc supplementation on growth was conducted by Brown et al in 2002 [4]. This review included studies with children until pre-puberty and studies from both developing and developed countries. Pooled results from 33 studies showed that zinc supplementation had a highly significant positive effect on linear growth (effect size = 0.350, 95% CI: 0.189, 0.511). In the latest review published by the same authors [3], more studies have been added to the previous (2002) analysis and the beneficial effect remained significant [effect size = 0.170 (95% CI: 0.075, 0.264)]. In contrast, another meta-analysis by Ramakrishnan et al. based on 43 studies found no significant effect of zinc supplementation on linear growth [effect size = 0.07 (95% CI: -0.03, 0.17)] in children < 5 years of age [5]. Our results from included studies from developing countries and with children < 5 years of age showed that preventive zinc supplementation (zinc ± iron) had a significant effect on linear growth [Effect size = 0.13 (95 % CI 0.04, 0.21)]. If we include results from four excluded studies from developed countries [23-26], the effect size comes to be 0.14 (95% CI 0.05-0.22) and if the results of studies with children > 5 years are included, the estimate becomes 0.15 (95 % CI 0.07, 0.23).

Our results are in concordance with that of Brown et al. 2009 [3] but at variance with the findings of Ramakrishnan et al 2009 [5]. What are the reasons for these contradictory findings and the differences between these reviews? The main differences were in inclusion and exclusion criteria. We and Ramakrishnan et al (5) included studies with children < 5 years, while Brown et al. (3) also included children until pre-pubertal age. Both Brown et al. (3) and we excluded studies where zinc was supplemented in fortified foods, while Ramakrishanan et al. (5) included these as well. Brown et al. (3) also excluded those studies where SD for change in length was not reported in the paper; however we and Ramakrishnan included those (5). Do these differences explain the reasons for contradictory results? If we include zinc fortified studies with our analysis (all countries), the estimated effect size becomes 0.15 (95 % CI 0.06, 0.20), which indicates that excluding zinc fortification studies did not significantly alter the results and direction of effect. Excluding children > 5 years also did not change the results significantly as shown above. We could not find any further explanation for the difference in results of our analysis and these reviews.

Presentation of results in terms of absolute difference in the growth increment is described in previous reviews. In the previous review by Brown et al. in 2002 (4) data were analyzed in two ways i.e. pooled weighted mean difference (results discussed above) and actual increase in length (cm) [4]. In the second analysis, they pooled results from 25 studies to get a net increment in length of 0.73 (±0.98) cm in zinc supplemented group compared to controls. These results were not however described for any particular dose or duration and the meta-views were also not provided to get an idea of the contribution of each study. On the other hand we have described pooled results for all the studies that reported absolute increment in length and also did a subgroup analysis for the most effective *dose* for a particular *duration*. This leads to an estimated effect size of 0.37 (±0.25) cm increase in linear growth in children <5 years, with a specific dose of *10 mg zinc/day* for a duration of *24 weeks* (Fig 3). The p-value for this estimate was 0.005 and quality grade for the pooled evidence was that of ‘moderate’ level. The most prominent contribution to this estimate comes from study by Umeta et al. in stunted children [45]. This is understandable, as zinc seems to have more prominent effect on growth in stunted compared to non-stunted children [4]. If results of this data set are omitted from analysis, the estimate becomes 0.36 (± 0.26) cm. In any case, keeping in mind the statistical significance, quality grade and specificity of estimate, this seems to be the most suitable input to LiST model, for an effect size estimate of zinc supplementation in prevention of stunting in children < 5 years of age in developing countries.

What does an extra gain of 0.37 cm means clinically? An average gain of this much in height would not be a huge effect but we need to take into account that that a single micronutrient would not be expected to result in such a substantial benefit at the first place. The results of this review confirms that preventive zinc supplementation indeed has an effect in promotion of growth of young children but this has to be connected with more comprehensive approaches that improve the diets of small children in general to get a more substantial effect. These efforts should especially focus on first two years of life and there should be a special attention to promote exclusive breastfeeding and practices of complementary feeding in addition to correcting micronutrient deficiencies. An analysis published in lancet undernutrition series in 2008 showed that education and counseling of caretakers in food-secure populations can improve growth in height (WMD 0.25; 95% CI: 0.01, 0.49) and providing complementary food, with or without education and counseling, can improve height in food insecure populations (WMD 0.41; 95% CI: 0.05, 0.76) [70]. In an updated analysis for this series, we have shown that provision of complementary food (±nutrition counseling) lead to an extra gain of 0.54 cm (±0.38) and education of mothers about complementary feeding can lead to an extra gain of 0.49 cm (±0.50) in the intervention group compared to control [71]. Thus, in order to get full advantage of relatively small benefit of correcting zinc deficiency, we must, at the same time, focus on interventions to improve complementary feeding and general nutritional status if the child. Future research should focus on strategies where correction of micronutrient deficiencies should be a part of a more general approach to improve the nutritional status of the child in general.

Do zinc and iron interact significantly when supplemented simultaneously? It has been demonstrated from several studies that iron and zinc have similar absorption and transport mechanisms [72]. Experimental studies have also shown that simultaneous supplementation of iron and zinc may inhibit zinc absorption in these cells especially at high ratios of iron to zinc [14,15,73]. A review by Walker et al. (64) on interaction of zinc and iron in supplementation trials showed that combined supplementation of zinc and iron did not affect the biochemical status of zinc; however the data were not clear regarding morbidity and growth outcomes. In our review, subgroup analysis excluding studies in which zinc was co-administered with iron showed an increase in overall effect size confirming the likelihood of interaction (Fig 2). When we separately pooled these data sets (zinc + iron), the pooled effect size showed a negative trend -0.10 [95 % CI -0.21, 0.01]. These results were significantly different from overall estimate (p=0.0007). These analyses strongly suggest that addition of iron decreases the positive effect of zinc supplementation on linear growth through potential interference with absorption or bio-availability. We have, therefore, presently restricted our recommendation for zinc supplementation to studies with zinc supplementation alone.

We used rigorous eligibility criteria for inclusion of studies. For example, the minimum period of supplementation should be ≥8 wk because shorter periods may be insufficient for detecting a linear growth response. Furthermore, we excluded studies of premature infants and those suffering from chronic disease or severe protein-energy malnutrition as zinc requirements of these children might differ considerably from those of unaffected children [74,75]. We also excluded studies in which zinc was supplemented in fortified food. Although tracer-element experimental studies had shown that zinc fortification of food increase total zinc absorption [76-78], relatively few community studies have found positive impacts of zinc fortification on serum zinc concentrations or functional indicators of zinc status [3]. There is also insufficient evidence for ideal food vehicle for zinc fortification and also interaction of zinc with other micronutrient when fortified in a single food [9].

	Preventive zinc supplementation seems to be a safe intervention. It has been suggested that high levels of zinc intake may interfere with normal iron and copper metabolism [76]. Although we did not specifically look at adverse effects, results from previous reviews showed that preventive zinc supplementation in physiological doses do not significantly affect the indicators of iron (i.e. hemoglobin and/or ferritin level) and/or copper metabolism [3].

Our review has certain limitations. As our recommendations are limited to zinc supplementation alone vs. control/placebo, these results may not be readily applicable to countries where there are on-going national supplementation programs of iron-folate, for example, India. We expect that policy makers will assess local contexts and conditions while considering the feasibility of zinc supplementation programs. We were unable to identify any significant predictor of substantial heterogeneity in the pooled data. There may be factors, not examined by us, that might explain the observed differences. These include initial HAZ score (prevalence of stunting), mean initial age, baseline zinc deficiency, gender and HIV prevalence [4,79-81]. Although there are relatively large number of studies and a funnel plot for zinc alone supplementation was relatively symmetrical (data not shown), there may be possible publication bias.

In conclusion, our review suggests that zinc supplementation has a positive effect on linear growth, especially when supplemented alone. Zinc supplementation in a dose of 10 mg/day for duration of 24 weeks led to an increase gain in length by 0.37 (± 0.25) cm among children < 5 years of age in developing countries compared to controls.

## Competing interests

We do not have any financial or non-financial competing interests for this review.

## Authors' contributions

Professor Zulfiqar A Bhutta developed the parameters for the review and secured support. Dr Aamer Imdad undertook the literature search, data extraction and wrote the manuscript under the supervision of Professor Bhutta.

## Supplementary Material

Additional File 1 Risk of bias for the included studies according to the latest recommendations of the Cochrane Handbook Click here for file
